# Modulation of integrin-linked kinase (ILK) expression in human oesophageal squamous cell carcinoma cell lines by the EGF and TGFβ1 growth factors

**DOI:** 10.1186/1475-2867-6-12

**Published:** 2006-04-27

**Authors:** Glenn A Driver, Robin B Veale

**Affiliations:** 1 School of Molecular and Cell Biology, University of the Witwatersrand, Johannesburg 2050, South Africa

## Abstract

**Background:**

Integrin-linked kinase (ILK) is a ubiquitously expressed protein kinase that has emerged as one of the points of convergence between integrin- and growth factor-signalling pathways.

**Results:**

In this study we identify the ILK isoform expressed in five human oesophageal squamous cell carcinoma cell lines of South African origin as ILK1, and demonstrate its cellular distribution. ILK expression, although similar in the majority of the cell lines, did show variation. Furthermore, the ILK expressed was shown to be catalytically functional. The effect of growth factors on ILK expression was examined. An increase in ILK expression, following EGF and TGFβ1 exposure, was a trend across all the five oesophageal carcinoma cell lines tested.

**Conclusion:**

These results suggest that growth factor modulation of ILK expression relies on the internalisation/recycling of growth factor receptors and stimulation of the PI3K pathway, which may have implications with regards to cell adhesion and tumourigenesis.

## Background

While responsiveness to growth factors is central to a host of normal cellular events, aberrant activity plays a key role in tumour development [1-3]. Among the numerous growth factors identified, epidermal growth factor (EGF) and transforming growth factor-1 (TGFβ1) are key players in neoplastic progression [2,4]. The action of EGF has been implicated in malignant transformation in squamous cancers of the bladder, breast and lung [5], whereas colon, gastric, endometrial, ovarian and cervical malignancies exhibit loss of response to TGFβ1 as a growth inhibitor [6,7]. Regarding carcinoma of the oesophagus in particular, reduced TGFβ1 has been correlated with depth of invasion, lymph node metastasis and poor prognosis [8].

In normal epithelial cells, appropriate cellular attachment is necessary for signalling pathways to be elicited [9,10]. More specifically, the response to growth factors can be potentiated by the integrin class of adhesion receptors through the integrin-associated protein, integrin-linked kinase (ILK) [1,7,11].

The ILK protein is a ubiquitously expressed serine/threonine protein kinase that is activated in response to PI3K stimulation [9,11-17]. Once activated, ILK couples integrins to downstream signalling pathways that are involved in the suppression of apoptosis and in promoting cell cycle progression [9,13,15,18-21]. The functional significance of ILK in malignancies is highlighted by the fact that ILK is overexpressed in several human tumours, including Ewing's sarcoma, primitive neuroectodermal tumour and medullablastoma [11,22,23]. Furthermore, ILK has been shown to induce an invasive phenotype in prostate and brain tumour cell lines [19,24].

Since our focus concerns oesophageal carcinoma, ILK expression is particularly intriguing since this tumour has a propensity to invade and metastasise. Carcinoma of the oesophagus has an extremely high incidence with high mortality and a poor prognosis [25-27]. It has the widest variation in incidence by geographical location of any neoplasm occurring in China, the Transkei region of South Africa, France and northern Iran [25-27]. In the cell lines under investigation, EGF is of particular relevance, stemming from the demonstration that these cell lines overexpress the EGF receptor [28]. It therefore becomes necessary to consider the effects of growth factors when examining the regulatory mechanisms of ILK expression. This report demonstrates novel data for the expression of ILK in five human oesophageal carcinoma cell lines and that this expression is modulated by EGF and TGFβ1.

## Results

### ILK1 in Human Oesophageal SCC cell lines (HOSCCs)

**Figure 1:**
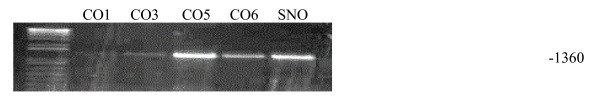
**RT-PCR analysis of ILK expression in HOSCCs**. RT-PCR was performed using specific primers co-amplifying ILK1 and ILK2. PCR products in the HOSCC cell lines WHCO1, WHCO3, WHCO5, WHCO6 and SNO separated on 2% agarose gels and stained with ethidium bromide. (A) An ILK band of 1360 bp was amplified across all five cell lines. 1 kb ladder in lane 1.

**Figure 2:**
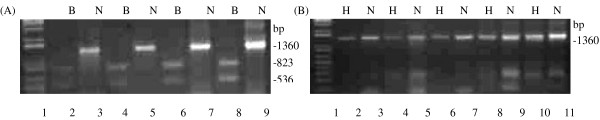
**Determination of ILK isoform/s in HOSCCs**. Amplified cDNA fragments were digested with either *Bam*H1 (B) or *Hinc*II (H) restriction enzymes. Non-digested (N) and digested fragments (B, H) were separated on 2% agarose gel electrophoresis and stained with ethidium bromide. (A) *Bam*H1 restriction analysis produced two fragments of 823 and 536 bp respectively whereas (B) *Hinc*II restriction analysis did not digest the 1360 bp ILK fragment. 1 kb ladder in lane 1.

RT-PCR analysis identified a prominent ILK fragment of 1360 bp in all the HOSCC cell lines (Figure 1), corresponding to the size of ILK in published data [1,29]. Furthermore, RFLP analysis showed the ILK, expressed by the HOSCCs, to be digested by *Bam*H1 to two fragments of approximately 823 and 536 bp respectively (Figure 2A). Digestion of the ILK fragment did not occur with the *Hinc*II restriction enzyme (Figure 2B) confirming that these oesophageal SCC cell lines only express the ILK1 isoform.

### ILK protein expression under standard culture conditions

**Figure 3:**
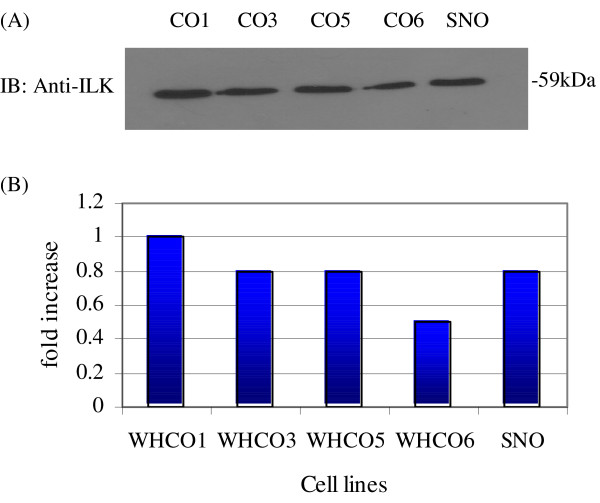
**ILK expression under standard conditions**. (A) Membrane associated cell lysates were resolved on 10% SDS-PAGE gels, blotted onto nitrocellulose filters and probed with anti-ILK antibody (1:1500). Each cell line produced a single anti-ILK reactive band of 59 kDa that varied in concentration. (B) Subsequent densitometric analysis utilising Labworks™ Image Acquisition and Analysis software (Labworks version 4.5) was performed on all cell lines to determine the relative levels of ILK expression under standard tissue culture conditions.

Immunoblotting analysis of Triton X-100 based extractions revealed a single 59 kDa band present in the membrane-associated fractions from the HOSCCs (Figure 3A). These results compare favourably with data obtained for ILK expression in colon and prostate carcinomas, as well as neuroblastoma, and melanoma cells [1,12,30], and therefore represent the first demonstration of ILK expression in human squamous carcinoma cell lines of the oesophagus.

Densitometric analysis revealed that ILK expression levels were comparatively similar across the WHCO3, WHCO5 and SNO cell lines (per μg of total cellular protein extracted). However, differences in expression were observed for cell lines WHCO1 (1.3 fold higher) and WHCO6 (1.5 fold lower) than the average (Figure 3B).

### Localisation of ILK in HOSCCs

**Figure 4:**
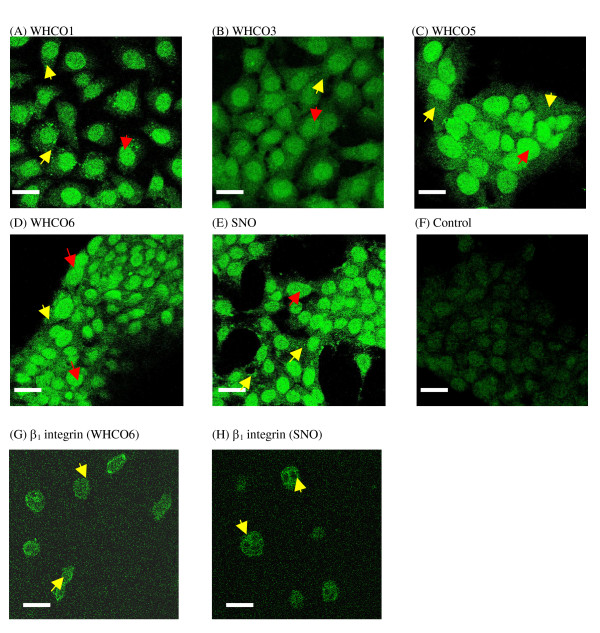
**Immunfluorescence localisation of ILK**. Under standard tissue culture conditions ILK localised to both cytoplasmic and nuclear compartments (red arrows). ILK was detectable at focal adhesions (yellow arrows) in the (A) WHCO1, (B) WHCO3, (C) WHCO5, (D) WHCO6 and (E) SNO cell lines. ILK was stained utilising an anti-ILK antibody (1:500). Bar, 10 μm.

**Figure 5:**
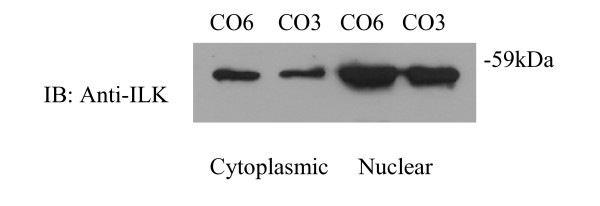
**ILK in nuclear fractions**. Membrane/nuclear cell fractions (10 μg/ml) were immunoblotted as before with anti-ILK antibody (1:1500). (A) ILK protein expression in the WHCO6 and WHCO3 cell lines in the cytoplasmic and nuclear fractions.

In addition to the expected cytoplasmic localisation, a large proportion of the cellular ILK was found to be present in the nucleus (Figure 4A–E, red arrows). Both nuclear and cytoplasmic localisation of ILK was corroborated by immunoblotting extracts of nuclear fractions (Figure 5, example WHCO6 and WHCO3). More typical of cytoplasmic ILK localisation was the observed focal adhesion association of ILK, implicating ILK in cell-ECM interactions (Figure 4A–E, yellow arrows). When analysed in conjunction with the β_1 _integrin subunit, it was noted that both ILK and the β integrin subunit followed a similar focal adhesion distribution pattern (figure 4G and 4H, yellow arrows) supporting the notion that ILK plays a significant role in cell-ECM adhesion processes.

### ILK expression following time-dependent exposure to EGF in oesophageal SCC cell lines

**Figure 6:**
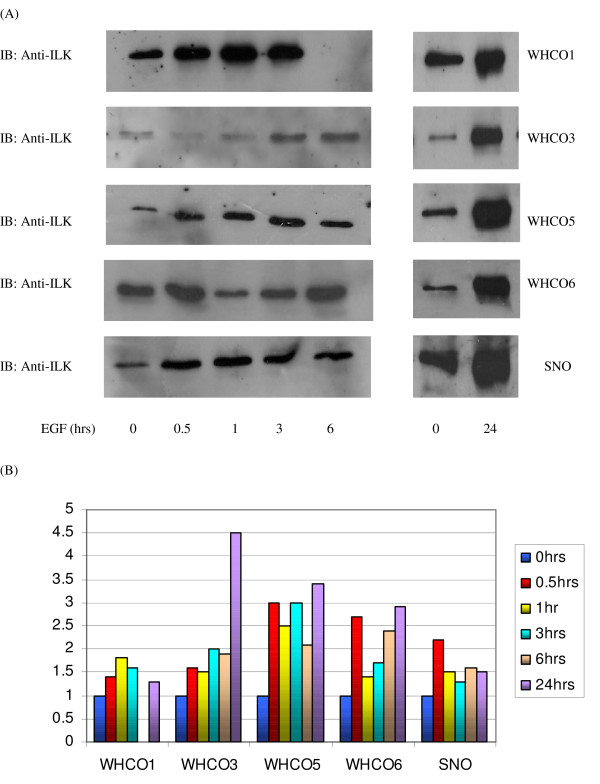
**ILK is catalytically active in HOSCCs**. ILK was immunoprecipitated using protein A sepharose beads (25 mg). MBP kinase assays were performed utilising 10 μCi [γ-^32^p] ATP and kinase reaction products were resolved on 10% SDS-PAGE subjected to autoradiography. (A) MBP phosphorylation in the WHCO1, WHCO3, WHCO5 WHCO6 and SNO cell lines. (B) Semi-quantitative ILK kinase activity established by densitometry of autoradiograms. The levels of MBP phosphorylated kinase products of WHCO1, WHCO3, WHCO5, WHCO6 and SNO. (C) The effect of 24 hours EGF and TGFβ1 exposure on ILK kinase activity in the WHCO1, WHCO3, WHCO5 and SNO cell lines. (D) Semi-quantitative ILK kinase activity established by densitometry of autoradiogams. Results are representative of three independent experiments.

Having established baseline data for ILK in HOSCC the effect of growth factors on ILK protein expression was examined. Exposure to EGF generally produced an increase in ILK expression in the five oesophageal carcinoma cell lines, although there was some variation in the magnitude of the increases. Similarities in ILK expression levels were observed between the WHCO3, WHCO5 and WHCO6 cell lines, whereas ILK levels in the WHCO1 and SNO cell line each responded somewhat differently to growth factor treatment (Figure 6A).

In the case of the WHCO1 cell line, highest ILK expression was noted after 1 hour EGF exposure where a fold increase of 1.8 fold was observed. This increase was maintained though to 24 hours where a 1.3 fold increase in ILK expression was seen. The WHCO3, WHCO5 and WHCO6 cell lines produced similar trends in ILK expression following time dependent exposure to EGF. ILK expression levels continued to increase following increased exposure to EGF in the WHCO3 cell line, reaching a substantial increase of 4.5 fold after 24 hours EGF treatment. Similarly, in the WHCO5 and WHCO6 cell lines, a large increase was observed following 0.5 hours EGF treatment of 3 and 2.7 fold respectively. Increased ILK expression levels remained constant and highest ILK expression was observed following 24 hours EGF exposure of 3.4 and 2.9 fold respectively (figure 6B).

The SNO cell line was somewhat outstanding in that this cell line elicited greatest ILK expression of 2.2 fold following 0.5 hours EGF exposure. Thereafter, a less substantial increase was noted at 1 hour of 1.5 fold, which was maintained through to 24 hours (Figure 6B).

### ILK expression following time-dependent exposure to TGFβ1 in oesophageal SCC cell lines

**Figure 7:**
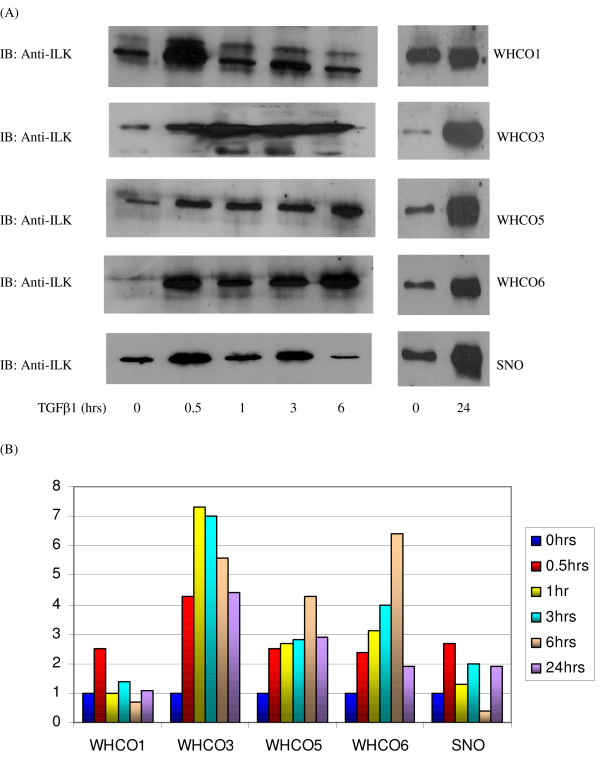
**Effect of EGF on ILK expression over time**. (A) Serum starved cells were treated with 10 ng/ml EGF for 0, 0.5, 1, 3 and 6 hours respectively and membrane associated fractions immunoblotted as before with a anti-ILK antibody (1:1500) in the WHCO1, WHCO3, WHCO5, WHCO6 and SNO cell lines. (B) Densitometric analysis of the expression levels of ILK in the HOSCCs demonstrated an increased trend in ILK expression, although variations in expression were noted at certain instances.

Following treatment with TGFβ1 produced an overall an increase in ILK expression (immunoblotting), not unlike the effect of EGF. However, variations were noted in the WHCO1 and SNO cell lines where fluctuations in ILK expression were observed (Fig. 7A). After 0.5 hours ILK expression increased by 2.5 and 2.7 fold in the WHCO1 and SNO cell lines respectively. By 1 hour ILK expression in WHCO1 cells returned to levels comparative to untreated fractions, whereas in SNO ILK expression had decreased. By 3 hours however, an overall increase of 1.4 (WHCO1) and 2 (SNO) had been attained. Surprisingly, a reduction was observed at 6 hours in comparison to the untreated fraction, 0.7 and 0.4 fold below for the WHCO1 and SNO cell lines respectively. Thereafter ILK expression levels increased after exposure for 24 hours by 1.1 (WHCO1) and 1.9 (SNO) fold (Figure 7B).

The WHCO3, WHCO5 and WHCO6 cell lines demonstrated a similar trend in increasing ILK expression in response to TGFβ1 treatment. The WHCO3 cell line produced a substantial increase in ILK expression reaching and maintaining a 7 fold increase over 3 hours exposure. Surprisingly, exposure to TGFβ1 for 6 hours did not produce a higher increase in ILK expression, although ILK expression was still 5.6 fold higher than the untreated control (Figure 7B). ILK expression continued to decrease at 24 hours TGFβ1 exposure but still producing a 4.4 fold increase. Exposure to TGFβ1 for 0.5 hours in the WHCO5 and WHCO6 cell lines caused an increase in ILK expression of 2.5 and 2.4 fold respectively, which was maintained continued to increase reaching 2.8 (WHCO5) and 4 (WHCO6) fold. ILK expression reached peak levels at 6 hours of 4.3 (WHCO5) and 6.4 (WHCO6) fold (Figure 7B). By 24 hours, ILK levels decreased producing a much reduced increase of 2.9 (WHCO5) and 1.9 (WHCO6) fold.

### EGF and TGFβ1 downregulate ILK Activity in HOSCCs

**Figure 8:**
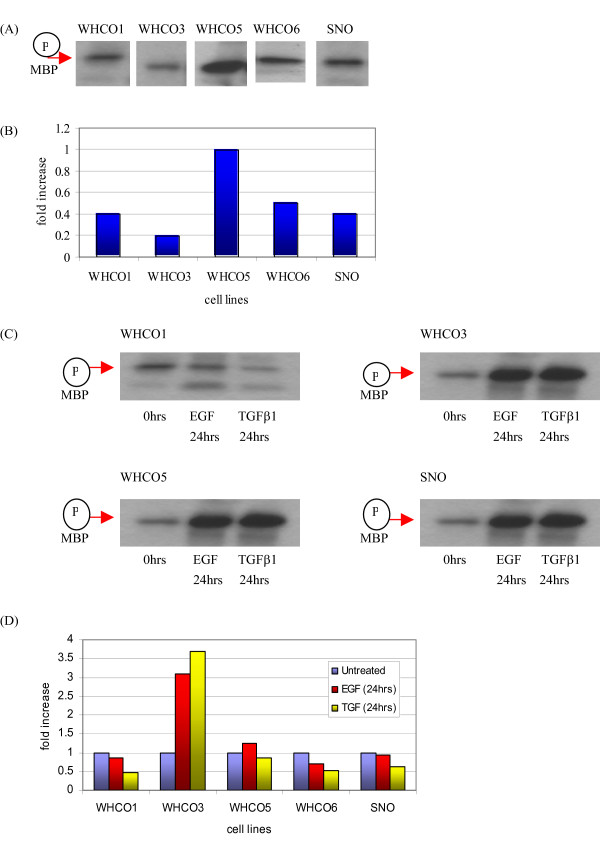
**Effect of TGFβ1 on ILK expression over time**. (A) Serum starved cells were treated with 10 ng/ml TGFβ1 for 0, 0.5, 1, 3 and 6 hours respectively. Membrane-associated fractions were resolved on 10% SDS-PAGE gels, blotted onto a nitrocellulose filter and probed with an anti-ILK antibody (1:1500) in the WHCO1, WHCO3, WHCO5, WHCO6 and SNO cell lines. (B) Densitometric analysis revealed that although expression levels of ILK were generally increased across the five HOSCC cell lines, fluctuations were observed in the WHCO1 and SNO cell lines.

Having determined the presence of ILK, it became necessary to assess the functionality of the catalytic domain. Furthermore, since growth factors were shown to stimulate ILK expression the effect of these growth factors on ILK activity was of interest. In this instance, myelin basic protein (MBP), which contains a β_1 _integrin peptide, was used to demonstrate the basal kinase activity of ILK. The five cell lines displayed a range of activities with the WHCO5 cell line exhibiting highest ILK activity (higher by 2 fold than average ILK activity) and the WHCO3 demonstrating much lower activity levels (2.5 fold lower than average) (Figures 8A and 8B). The remaining three cell lines (WHCO1, WHCO6 and SNO) produced a similar pattern of activity. These data demonstrated that ILK activity levels show some variation across the five moderately differentiated oesophageal SCC cell lines examined.

Interestingly, it was observed that treatment of cell cultures with 10 ng/ml EGF and TGFβ1 for a period of 24 hours resulted in a decline in ILK kinase activity of 1 to 3 fold in cell lines WHCO1, WHCO6 and SNO (established by densitometry, figure 8C and 8D). However, paradoxical results were obtained in the case of the WHCO5 cell line, where treatment with TGFβ1 reduced ILK activity, whereas EGF augmented ILK activity. The addition of growth factors had the greatest effects on the WHCO3 line, which showed a dramatic increase in activity to both EGF (3 fold) and, TGFβ1 (3.5 fold). Although TGFβ1 treatment had a similar effect to that of EGF, its effect on ILK activity was more profound. When considering growth factor effects upon ILK expression and activity, it became apparent that increased ILK expression induced by growth factors did not necessarily imply a similar response in ILK activity.

## Discussion

Despite data being available for ILK expression in a variety of tumour types (prostate, breast, and colorectal, [11,12,19,22,23], the same cannot be said for carcinoma of the oesophagus. Moreover, since the cell lines examined here are known to overexpress the EGF receptor [28], determining the effects of growth factors on ILK expression is pertinent to our understanding of the adhesion/signalling role of ILK in the moderately differentiated class of oesophageal SCC.

The two mRNA isoforms of ILK (ILK1 and ILK2), share similar structural and functional properties but, whereas ILK1 is expressed in all normal tissues, ILK2 has only been identified in two highly metastatic fibrosarcoma and melanoma cell lines [1]. Here we established that the ILK1 isoform predominates in HOSCCs, and is catalytically functional. In addition to ILK demonstrating a typical focal adhesion localisation, ILK was also observed in the nuclear and cytoplasmic compartments. The nuclear distribution of ILK is somewhat surprising but has been demonstrated previously and may be attributable to an impairment of the ILK association with either PINCH or caveolin [21,23]. These data therefore provided the baseline for examining the effects of growth factor on ILK protein expression in HOSCC cells.

With the exception of the WHCO1 and WHCO6 cell lines, the expression levels of ILK were relatively similar among the WHCO3, WHCO5 and SNO cell lines (see figure 3B). However, since ILK expression levels were not uniform across all five HOSCCs, being somewhat higher in the WHCO1 cell line and lower in the WHCO6 cell line, we could not consider the observed ILK expression as peculiar to the moderately differentiated oesophageal phenotype.

ILK acts as a molecular scaffold during a host of signalling events, mediating crosstalk between integrins and the growth factor response [11], thus eliciting downstream events that impinge upon vital aspects of cellular function [10,31]. Although many of the signals elicited by ILK are a direct result of growth factor signalling, the effect of growth factors on ILK protein expression and kinase activity has not been considered to any great extent. In these HOSCCs, the general response of ILK to both EGF and TGFβ1 treatment was an increase in ILK protein expression levels over the duration of growth factor exposure (see figures 6B and 7B). It is likely that this stimulation occurs via activation of the PI3K pathway, which has been shown to be activated in response to these growth factors [32,33]. The only other report of EGF and TGFβ1 effects on ILK expression, showed stimulation of ILK expression in a human melanoma cell line following exposure to EGF and TGFβ1 for 24 and 48 hours [1]. However, since a rapid response in ILK expression to both EGF and TGFβ1 by most cells has been shown previously [4,34], the present study concentrated on shorter exposure times in addition to the longer exposure times.

The noted variations in ILK expression among the cell lines suggest that a receptor recycling mechanism occurs in response to these growth factors depending on the time exposure. In the instances where ILK expression decreased abruptly, the decrease could be attributable to a reduction in the number of receptors at the membrane as a result of receptor internalisation [35]. It is widely accepted that, although EGFR localises primarily at the cell surface, they constantly recycle through the cell [36]. Furthermore, rates in recycling can be accelerated as a consequence of EGF ligand-binding [34,36-43]. It is highly probable that the noted fluctuations in ILK expression in the WHCO3, WHCO5 and SNO cell lines are attributed to this receptor internalisation/recycling process where lowered EGF signalling occurs as a result of EGFR reduction at the plasma membrane. A direct consequence being reduced ILK activated due to reduced PI3K signalling.

Likewise, the receptors of TGFβ1 also undergo recycling both in the presence and absence of ligand activation [44]. Although scarce, early reports suggest that internalised receptors are either rapidly replaced or recycled back to the cell membrane [45,46]. More recently, [46] showed that in lung epithelial cells TGFβ1 receptors do indeed recycle and degrade in a clathrin-dependent approach, but in a manner that is not dependent on ligand stimulation. It is possible that ligand stimulation may be required in the case of HOSCCs and the noted fluctuations following TGFβ1 exposure may be explained by receptor recycling.

To determine whether growth factors induced a similar response in both ILK expression and activity in HOSCCs, ILK kinase assays were conducted in the presence of EGF and TGFβ1. Given that the greatest increase in ILK expression was noted following 24 hours growth factor exposure, cells were exposed to growth factors for a similar period of time. With the exception of the WHCO3 cell line which showed a marked increase in ILK kinase activity, the general trend was a decline in ILK kinase activity over the other cell lines following growth factor exposure (see figure 8D). This data is surprising since [24], have previously shown that a rapid stimulation of ILK activity by insulin and PDGF occurred in intestinal epithelial cells.

The substantial increase in ILK activity in the WHCO3 cell line could be attributed to decreased PTEN since TGFβ1 has been shown to downregulate PTEN mRNA in keratinocytes [47]. In contrast, EGF and TGFβ1 may be stimulating PTEN activity in the WHCO1, WHCO6 and SNO cell lines. Due to the increased EGFR that have been reported with regards to the oesophageal SCC lines, one could also argue that the cell lines WHCO1, WHCO6, and SNO are at an upper limit of growth factor response. Thus, growth factor treatment of these cell lines would not elicit any further cellular responses, a consequence being that ILK would not be activated, accounting for the low levels of ILK kinase activity observed in these cell lines. This speculation is supported by the suggestion that in fibroblasts higher TGFβ1 concentrations results in inhibition of both cell migration and proliferation [48].

While the cellular expression of ILK in HOSCCs is comparable to that of other cell types, particularly noteworthy is that ILK expression in HOSCCs appears to be dependent on the receptor recycling of both the EGF and TGFβ1 receptors. EGF/TGFβ1 has been shown to result in a rapid, marked increase in ILK expression, and since it is known that EGFR overexpression occurs in these HOSCCs, it is plausible that a constitutive increase in ILK expression exists in these HOSCCs. A direct consequence of EGF/TGFβ1 stimulation of the ILK pathways could thus be decreased cellular adhesion due to impaired integrin-mediated signalling, or a stimulation of mitogenic pathways such as MAPK thereby promoting transformation.

## Materials and methods

### Cell lines

Five South African moderately differentiated human oesophageal squamous carcinoma cell lines WHCO1, WHCO3, WHCO5, WHCO6 [28] and SNO [49] were obtained from the Cell Biology Laboratory, School of Molecular and Cell Biology, University of the Witwatersrand. Medium was removed and cells washed twice with pre-warmed (37°C) phosphate buffered saline (PBS). Cells were harvested using 0.05% (w/v) trypsin and 0.01% (w/v) EDTA. Cell lines were cultured in 10 cm Nunc™ tissue culture dishes with Dulbecco's Modified Eagles Medium (DMEM)/Hams F12 (3:1), supplemented with 10% Foetal Calf (or bovine) Serum (FCS or FBS). Cultures were maintained in a humid, 37°C incubator, 5% carbon dioxide (CO_2_) atmosphere.

### ILK amplification by reverse transcription polymerase chain reaction (RT-PCR)

Cells were grown to 80% confluence in sterile 10 cm Nunc™ tissue culture dishes and total RNA isolated using TRIzol^® ^solution (GibcoBRL). For reverse transcription, 2μg of total RNA was converted into cDNA by MMLV reverse transcriptase at 37°C for 1.5 hours in a 25μl RT reaction. Briefly, PCR amplification mixtures contained cDNA, ILK antisense primer (5'-CTCGAGCTACTTGTCCTGCATCTTCTCAAG-3'), sense primer (5'-GAATTCGTATGGACGACATTTTCACTCAGTGC-3') and Expand High Fidelity^PLUS ^Taq (Roche, SA). Thermal cycle conditions included denaturation at 95°C for 10 minutes, and 30 cycles between 95°C for 15 seconds, 50°C for 1 minute and a 2 minute extension step at 72°C, followed by a final elongation at 72°C for 10 minutes. RT-PCR products were visualised on 2% agarose gels following ethidium bromide (EtBr) staining.

### Restriction Fragment Length Polymorphism (RFLP) analysis of amplified ILK cDNA

ILK RT-PCR products were purified using a QIAGEN^® ^QIAquick Purification Kit. Thereafter, restriction digests were performed by the addition of *Bam*H1 and *Hinc*II restriction enzymes to purified ILK products, and incubated for 1.5 hours at 37°C. Subsequent digestions were cleared of protein by the addition of phenol/chloroform and centrifuged at 6000 rpm for 2 minutes and aqueous phase transferred to a sterile tube. Both 0.7 M EtOH and 3 M NaAc were added and allowed to precipitate DNA for 12 hours at -20°C. Samples were centrifuged at 6000 g for 20 minutes, the supernatant decanted, and the DNA pellet resuspended in dH_2_O. Products were visualised on 2% agarose gels following EtBr staining.

### Antibodies

Polyclonal rabbit anti-ILK antibody (Zymed^®^, USA) and polyclonal horseradish peroxidase (HRP)-bound anti-rabbit secondary antibody (Separations, SA) antibodies were used for immunoprecipitation and immunoblotting procedures. Polyclonal rabbit anti-ILK antibody and a fluoroscine isothiocyanate (FITC)-conjugated anti-rabbit secondary antibody (Chappel, USA) were used for indirect immunofluorescence.

### Indirect immunofluorescence

Cells grown to 80% confluency and seeded onto sterile glass coverslips were fixed with 4% paraformaldehyde and permeabilised in 0.25% Triton X-100. Exposure to anti-ILK (1:500) followed by incubation with an anti-rabbit Fluoroscine Isothiocyanate (FITC)-conjugated anti-rabbit antibody (1:1000) for ILK. Slides were viewed under a Zeiss LSM 410 confocal microscope (FITC excitation 490, emission 525).

### Preparation of nuclear cell lysates

Nuclear extractions were performed utilising the CelLytic™ Nuclear™ Extraction Kit (Sigma, USA), which is based on a hypotonic buffer treatment followed by a high salt buffer release of nuclei.

### Treatment of oesophageal carcinoma cell lines with EGF and TGFβ1

To examine growth factor effects on the WHCO1, WHCO3, WHCO5, WHCO6 and SNO HOSCC cell lines, cell cultures were first serum starved for a period of 16 hours. Cells were then treated with either 10 ng/ml EGF or TGFβ1 for a period of 0.5, 1, 3, 6 and 24 hours respectively. Experiments were repeated on three separate occasions.

### Preparation of membrane cell lysates

Cell cultures were washed in PBS containing 20 mM phenyl-methyl-sulphonyl fluoride (PMSF)/Aprotinin (Trasylol^® ^Bayer, SA) stock solution. The cell suspension was centrifuged at 1000 g for 2 minutes. The supernatant was decanted, and the cell pellet resuspended in an extraction buffer (containing 50 mM Tris, 150 mM NaCl, 1 mM CaCl_2_, 1 mM MgCl_2 _and 0.01% aprotinin pH 8.5) and incubated for 2 hours on ice. Thereafter, samples were centrifuged at 6000 g for 10 minutes. The resulting cell lysates were stored at -70°C. Total protein content was determined using the Bradford assay [50] using the protocol from [51].

### Immunoblotting

Cell lysates (10 μg) were electrophoresed through 10% SDS-PAGE gels according to Laemmli, (1970), transferred to Nitrobind nitrocellulose transfer membrane (MSI, USA). Nitrocellulose membranes were blocked with a casein-based blocking buffer (BLOTTO), incubated in polyclonal anti-ILK antibody (1:1500), followed by incubation in HRP-bound secondary anti-rabbit antibody (1:2500). Detection was carried out with the Supersignal^® ^West Pico Chemiluminescent kit. The experiment was performed on three separate occasions.

### Immunoprecipitation

Cell lysates (100 μg) were incubated with 2 μg anti-ILK antibody for 12 hours at 4°C. The resulting immune complexes were incubated for 4 hours at 4°C with protein A sepharose (Sigma, SA). Immune complexes were captured by centrifugation and immunoprecipitated ILK resolved on 10% SDS-PAGE gels, transferred to nitrocellulose filter and probed with an anti-ILK antibody (1:1500). Detection was carried out with the Supersignal^® ^West Pico Chemiluminescent kit (Pierce, USA).

### ILK kinase activity

Immunoprecipitated ILK was incubated in a kinase reaction buffer (50 mM Hepes, 10 mM MnCl_2 _and 10 mM MgCl_2 _pH7), 10 μCi [γ-^32^P] ATP (Amersham, UK) and 10 μg myelin basic protein (MBP) (Sigma, USA) for 2 hours at 30°C with gentle agitation. The reaction was terminated by the addition of an equal volume of 2 × laemmli buffer and incubation at 95°C for 5 minutes. Phosphorylated MBP products were recovered by centrifugation at 6000 g for 10 minutes and resolved on 10% SDS-PAGE, following Coomassie Blue staining, destaining, and rinsing in dH_2_O and 10% ethanol respectively. Gels were subsequently dried on a slab gel drier SE1160 (Hoefer, San Francisco). Phosphorylated MBP was detected via autoradiography.

### Densitometric analysis

Densitometric analysis of the western blot and kinase assay x-ray plates were used for semi-quantitative comparison of ILK protein levels by using the area under the peak. Expression levels were represented as a percentage of the maximum per 10 μg of protein. In interpreting the growth factor data, we considered a 1.2 fold increase (equivalent to 20%), to be an appreciable increase in ILK expression levels. All subsequent fold increases are compared to an untreated control, unless otherwise stipulated.

## Authors' contributions

GAD and RBV contributed equally to the conception and design of the study. GAD performed all experimental work. RBV participated in the coordination of the study. Both authors read and approved of the final manuscript.
